# Bioaccumulation of Some Metals and Metalloids in Laughing Gulls (*Leucophaeus atricilla*): Increases in Mercury and Decreases in Selenium from 2019 to 2022/2023

**DOI:** 10.3390/toxics11121007

**Published:** 2023-12-09

**Authors:** Joanna Burger, Stephanie Feigin, Alinde Fojtik, Amanda Dey, Kelly Ng

**Affiliations:** 1 Division of Life Sciences, Rutgers University, 604 Allison Road, Piscataway, NJ 08854, USA; kelng@dls.rutgers.edu; 2 Environmental and Occupational Health Sciences Institute (EOHSI), Piscataway, NJ 08854, USA; 3 Environmental Science Graduate Program, Rutgers University, New Brunswick, NJ 08903, USA; stephaniefeigin@gmail.com; 4 Ecology and Evolution Graduate Program, Rutgers University, New Brunswick, NJ 08903, USA; 5 Wildlife Restoration Partnerships, 109 Market Lane, Greenwich, NJ 08323, USA; 6 Southeastern Cooperative Wildlife Disease Study, University of Georgia, 589 D.W. Brooks Drive, Athens, GA 30677, USA; afojtik@uga.edu; 7 109 Market Lane, Greenwich, NJ 08323, USA; dey.amanda@gmail.com

**Keywords:** gulls, bioaccumulation, horseshoe crab eggs, Hg, Se, food chain

## Abstract

The elements in blood normally reflect the levels in prey, indicating a recent exposure. Laughing gulls (*Leucophaes atricilla*) eat mainly horseshoe crab eggs (*Limulus polyphemus*) in the spring in Delaware Bay, New Jersey. The levels of arsenic (As), cadmium (Cd), chromium (Cr), lead (Pb), mercury (Hg), and selenium (Se) in the blood of laughing gulls foraging on crab eggs were examined in Delaware Bay to provide information on a species that is normally a generalist, and to determine if the levels of these elements were similar in 2019 and 2022/2023, were intercorrelated, and were related to those in crab eggs. Hg increased from 2019 (136 ± 31 ng/g) to 2022/2023 (473 ± 75 ng/g), while Cd and Se decreased. There were some significant correlations among elements and a close relationship between the element levels in blood and those in crab eggs collected in the same month (except for As). The levels differed between laughing gulls and three species of shorebirds for As and Cd. The elements in the blood of gulls and shorebirds should be similar because they eat mainly the same eggs in the same places. A significant proportion of laughing gull blood samples had levels of Hg and Se that were above the levels associated with adverse effects, which requires further examination.

## 1. Introduction

Most birds acquire heavy metals, metalloids, and other contaminants through food or water. Aquatic birds are excellent bioindicators of environmental contamination because they feed on organisms in water, which is a major pathway for exposure to contaminants, particularly direct exposure from point-source pollution and runoffs [1,2]. Foraging and prey types are thus important aspects that contribute to understanding contamination in birds. The foraging behavior and prey type, abundance, and availability are important aspects that directly affect fitness in birds. Within this context, some species are normally generalists, while others are specialists. Under unique circumstances, several species that might otherwise have a varied diet can feed on the same prey item for an extended period. For example, when salmon are spawning, bears and gulls feed almost exclusively on salmon, as they are concentrated and dense in spawning streams [3]. Similarly, in the spring in Delaware Bay, and in some other estuaries along the Atlantic coast of North America, there is a massive spawning of horseshoe crabs (*Limulus polyphemus*), usually corresponding in time to the spring migration of shorebirds and the local breeding of laughing gulls. The abundant crab eggs from this spawning result in an excess of eggs on the surface of the sand and mud and on the windrows rolling in the surf, which are eaten by many species [2,4,5]. Horseshoe crab eggs also provide a food base for other species, including hatchling terrapins and small fish [2].

Shorebirds foraging on these eggs have garnered most of the attention concerning these phenomena, because the species migrating through Delaware Bay in the spring are some of the bird species that are declining at the highest rates of any avian species [6,7,8,9,10]. Shorebirds, however, are not the only species consuming large quantities of horseshoe crab eggs. The eggs are also eaten by gulls, including laughing gulls (*Leucophaeus atricilla*), herring gulls (*Larus argentatis*), and great black-backed gulls (*Larus marinus*), as well as egrets and even grackles (*Cassidix major*). However, the spectacle of thousands of laughing gulls (*Leucophaeus atricilla*) foraging in dense masses, sometimes forming swaths 3–4 m wide along the shore, is equally as unusual as the shorebirds, and the gulls are feeding on the same horseshoe crab eggs.

In this paper, we examined the levels of several metals and metalloids in the blood of laughing gulls foraging on horseshoe crab eggs along Delaware Bay beaches in early May. We tested the hypotheses that (1) there are no inter-year differences in element (metal and metalloid) levels, (2) elements are not correlated with one another in laughing gulls, and (3) there is no relationship between the levels in the blood of gulls and those in horseshoe crab eggs. Based on earlier work [11], we expected that the metal levels in bird blood would relate to the levels in the eggs of crabs. Since the levels of some elements such as selenium (Se) are homeostatically regulated in the blood, we expected the levels in the blood to be similar to those of other species that have been examined [12,13]. Considerable attention has been devoted to the temporal and spatial patterns of metal levels in shorebirds in Delaware Bay over several decades [4,11,13,14,15], but there are no data for the gulls.

From mid-April to late May or June, laughing gulls forage on the beaches from Villas north to Reeds Beach in Delaware Bay (New Jersey, USA). Lower numbers forage on the beaches farther north on the New Jersey side of the Bay. In mid-May, the number of laughing gulls begins to decline as they initiate incubation across the Cape May peninsula in the Stone Harbor marshes, which is the largest colony in New Jersey (and in the northeast [16]). Unlike the shorebirds, however, the gulls are not gaining weight in preparation for a long northward migration [5,17], but female laughing gulls are building up stores to lay eggs and both sexes are maintaining weight for normal breeding activities. Laughing gulls are generalists, however, and normally forage on a wide range of foods, from natural shoreline fish, invertebrates, and carrion to refuse at dumps and handouts at parking lots [15,18].

While the shorebirds make very long migrations and stop at Delaware Bay to refuel on their way to breed farther north [19,20,21,22,23], laughing gulls nest nearby in salt marshes along New Jersey’s Atlantic coast and migrate south only to the Caribbean or Gulf Coast [18]. The shorebirds that stop over at Delaware Bay remain for only two to three weeks [24,25], while laughing gulls are present throughout the horseshoe crab spawning season. Horseshoe crab abundance and egg availability are linked to shorebird survival and fitness [26,27,28,29]. One species of shorebird, the red knot (*Calidris canutus rufa*), is endangered in Canada and federally threatened in the United States [30,31]. During the shorebird stopover on Delaware Bay, they eat almost exclusively the eggs of horseshoe crabs [32,33], making it possible to compare the levels of metals in their blood (e.g., recent exposure) to the levels of these same metals in their prey (crab eggs). The gulls and shorebirds consume the same prey for days or weeks, which may equalize their exposure to contaminants. Indeed, the mean levels of different metals and metalloids in the blood of shorebirds are correlated with the mean level in crab eggs [11], but metal levels have not been examined in the blood of laughing gulls.

## 2. Approach and Methods

The overall protocol was to collect blood from laughing gulls in the mid-period of intense foraging (mid-May) on horseshoe crab eggs at the high tide line. We examined arsenic (As), cadmium (Cd), chromium (Cr), lead (Pb), mercury (Hg), and selenium (Se) in gull blood from 2019 and 2022/2023. Blood was placed in a cooler and frozen until it was analyzed at the Environmental and Occupational Health Sciences Institute at Rutgers University. All methods and sample collections were approved by the Rutgers University Institutional Animal Care and Use Committee (#92-036, renewed every three years), appropriate state and federal permits were obtained, and the study conformed to the animal welfare and research guidelines provided by the Ornithological Council of the US [34].

### 2.1. Study Species and Study Site (Delaware Bay, New Jersey, USA)

Laughing gulls nest in colonies of up to 25,000 pairs on sandy or rocky shores, and in salt marshes along the Atlantic and Gulf Coasts of North America, as well as on some Caribbean islands, the Gulf of California, and the Pacific Coast of Mexico [18,35]. A significant proportion of the North American breeding population is located on the New Jersey coast. Laughing gulls breeding in New Jersey generally move south to Florida or the Caribbean islands in the winter, returning in April. They are generalists in their foraging behavior and foraging habitats, as well as in their food items [16]. Laughing gulls eat fish, insects, other invertebrates, berries, garbage, and carrion; they follow boats, dive for fish, “hawk” for flying insects, catch food thrown by beachgoers or fishermen, and also pirate food from other species [17,36,37,38]. Thus, under normal circumstances, their foods are so variable that examining the bioaccumulation from specific prey is impossible.

**Figure 1:**
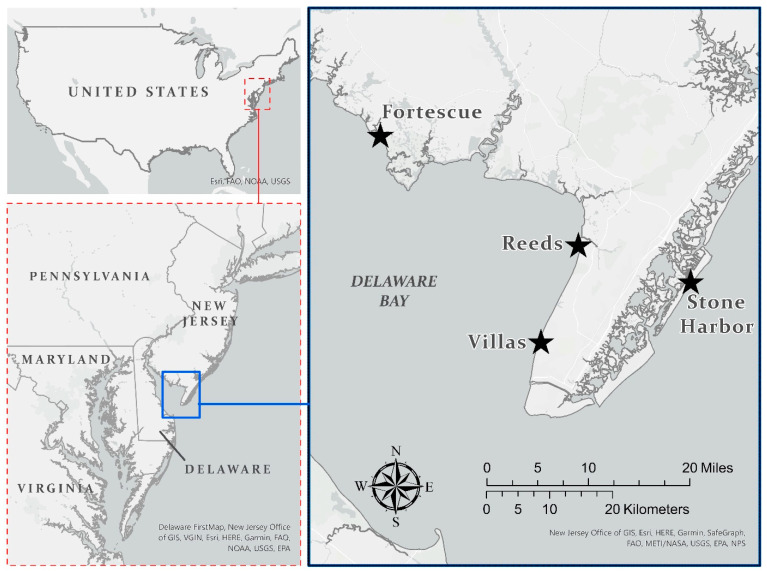
Map of Delaware Bay (New Jersey), with the sites where blood from laughing gulls was sampled, as well as Stone Harbor, where laughing gulls nest. The insert shows the location of Delaware Bay on the Atlantic Coast of North America.

Water from the Delaware River flows into the Delaware Bay, which is bordered by the states of New Jersey and Delaware. The water flows through the bay into the Atlantic Ocean (Figure 1). The bay ranges in width from 18 to 42 km, with a tidal range of about 1.3 m that provides extensive tidal flats. The Delaware Estuary is one of the world’s largest oil-refining ports [2]. Pollutants flow into the river and bay from industries, agriculture, and residential communities. In the 1990s, the US Environmental Protection Agencies established the Estuaries Program to characterize the pollutants and conditions of estuaries in the USA; As, Cr, Pb, and Hg were some of the primary contaminants of concern in Delaware Bay [2]. Recently, some fish known to be consumed by people were found to still be above the recommended consumption rates [39]. Salt marshes and sandy beaches fringe the bay, providing ideal habitats for the horseshoe crabs to spawn on the sandy beaches, laying thumb-sized clutches of eggs below the sand from mid-April to June [5].

### 2.2. Collection Methods

Laughing gulls (n = 46) were captured using a cannon net in May 2019 and 2022/2023 during the shorebirds’ northward migration through Delaware Bay. Birds were netted while feeding on crab eggs at the water line or roosting on nearby beaches. Thus, only gulls that fed on crab eggs were likely to be captured. Although the shorebirds that the gulls feed with were fattening up to fuel their continued migration north [27,33,40,41], the gulls were foraging to support their breeding that had already begun. Reproductive activities are energetically costly. The gulls nest only a few km away in a salt marsh colony in Stone Harbor, New Jersey. The netted gulls were processed, measured, and weighed, and blood was collected [11,14]. Less than 100 uL of blood was drawn from the cutaneous ulnar vein, using a 26G ½ inch needle to nick the vein. The blood was immediately drawn into heparinized capillary tubes, and the capillary tubes were then placed in non-additive glass vacutainers to prevent breakage. Much less than 1% of the body weight of blood was collected from each gull. In the field, the blood samples were placed in a cooler with ice and then stored at −20 °C for later processing.

### 2.3. Chemical Analysis

The samples were processed and digested at the Environmental and Occupational Health Sciences Institute (EOHSI) and analyzed in Nelson Hall at Rutgers University. The procedures described here are from Burger et al. [4,11,13,39] and Tsipoura et al. [24]. Operations in separate laboratories are advisable to prevent any cross-contamination. The containers and equipment were washed and rinsed (with a 10% HNO_3_ solution and deionized water) before any analyses. Blood from each bird was individually homogenized and digested in 70% TraceMetal Grade nitric acid (Sigma-Aldrich, St. Louis, MO 68178 USA) in a microwave (MDS 2000 CEM) (CEM Ltd., Holtsville, NY 11742, USA). The total Hg was analyzed using a Perkin Elmer FIMS-100 mercury analyzer (PerkinElmer Inc., Waltham, MA 02451 USA) with an instrument detection limit of 0.02 ng/g. The other metals were analyzed with a Perkin Elmer 5100 flameless atomic absorption spectrometer (Zeeman correction) (PerkinElmer Inc., Waltham, MA 02451 USA). The instrument detection limits were 0.02 ng/g (ppb) for As and Cd, 0.08 ng/g for Cr, 0.15 ng/g for Pb, and 0.02 ng/g for Se [11]. Standards, reference material (from NIST), spiked specimens, and blanks were analyzed along with the samples to evaluate the analytical control and accuracy. The reference material DORM-2, “Dogfish Muscle Certified Reference Material for Trace Metals” from the National Institute of Standards and Technology (NIST), was used for cold vapor atomic absorption spectroscopy (Hg). The standard reference material (SRM) 1640, “Trace Metals in Natural Water”, was used for a Zeeman graphite furnace atomic absorption spectroscopy quality control evaluation. The recoveries ranged from 85% to 115%.

### 2.4. Statistical Analysis

We used non-parametric procedures (Kruskal Wallis test, PROC NPAR1WAY [42]) to determine the differences in the heavy metal levels among gulls and three species of shorebirds. Kendall tau correlations were used for determining the relationships among metal levels in laughing gull blood. We used these non-parametric tests because they are more conservative and are best suited for small datasets that are not normally distributed [43]. The concentrations are expressed in parts per billion (ppb = ng/g) on wet weight. We accepted *p* < 0.05 as significant.

## 3. Results

**Figure 2:**
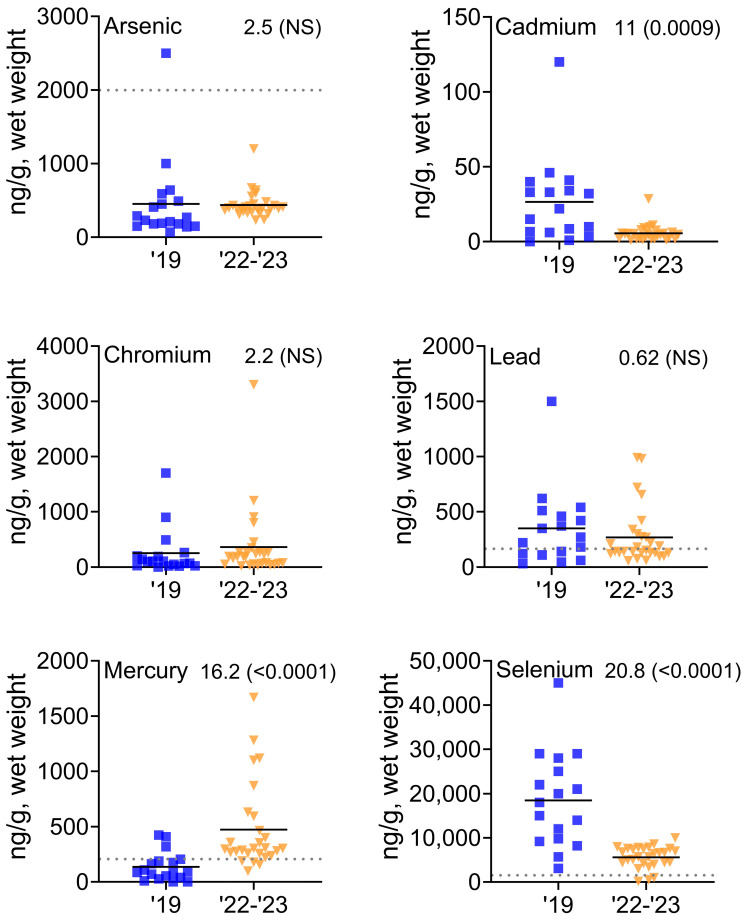
Distribution of the metals and metalloids in whole blood of laughing gulls foraging on horseshoe crab eggs in Delaware Bay as a function of year (2019, 2022/2023). Each point equals one gull; there is some overlap among the points; and the horizontal line = the arithmetic mean. The dotted line equals the lowest effect levels in birds (see Section 4). Years are compared with Kruskal–Wallis chi squared statistic and *p*-values are shown for each element.

Our overall goal was to examine the levels of As, Cd, Cr, Pb, Hb, and Se in the blood of laughing gulls. The levels of Cd were the lowest, and the levels of Se were the highest overall (Table 1). Cd was an order of magnitude lower than all the other metals, and Se was an order of magnitude higher than most of the other metals (Table 1). There were significant yearly differences in the levels in the blood of laughing gulls from 2019 to 2022/2023; Hg increased significantly, while Cd and Se decreased significantly (Table 1, Figure 2).

While means provide a useful measure, a visual depiction provides additional information on the variation and outliers (Figure 2). There was a great deal of variation in all the metals among individuals. There were several outliers, which suggests that some gulls may be acquiring metals in different places along the shore.

Our second hypothesis was that there were no correlations among metals. In other words, we aimed to examine whether gulls that had high levels of one element tended to have high levels of other elements (Table 2). There were positive correlations in the blood of laughing gulls between As and Cr, and As and Pb; between Cd and Cr, Pb, and Se; and between Cr and Pb. There were significant negative correlations between Hg and Cd, Pb, and Se. The negative correlation between Hg and Se is significant because of the potential mitigation relationship between these metals (see Section 4).

**Figure 3:**
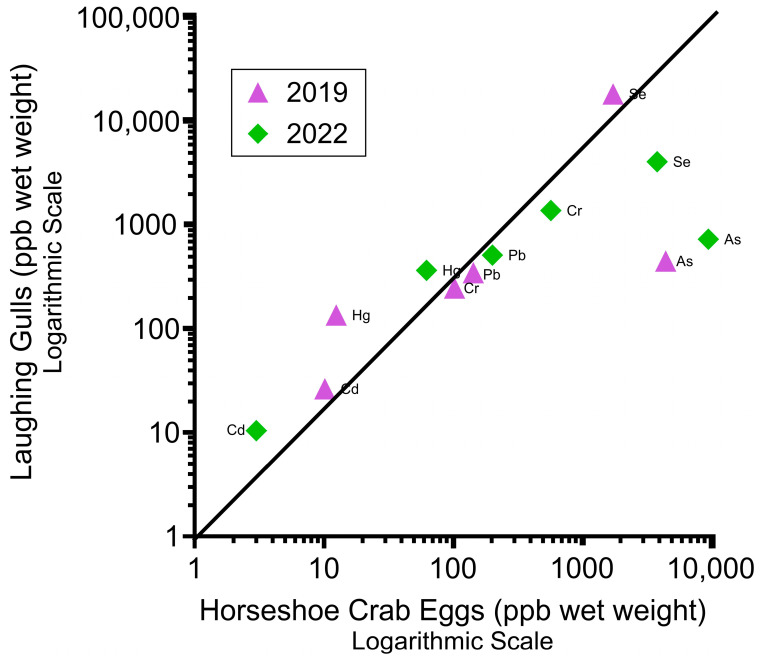
Relationship between the mean levels of elements in the blood of laughing gulls (n = 43) compared to horseshoe crab eggs for 2019 and 2022. The line connects equal concentration values. Data on horseshoe crab eggs are from Burger et al. [39] and Burger [13,44]. Horseshoe crab egg samples each contained about 300 eggs (n = 34 in 2019; n = 18 in 2022). Units are ng/g (ppb wet weight).

Our third hypothesis was that there was no relationship between the levels of metals in the blood of laughing gulls and those in horseshoe crab eggs from the same beach (Figure 3). Figure 3 shows the relationships between the mean levels of metals and metalloids in the blood of laughing gulls (in two time periods; 2019 vs. 2022/2023) and the mean levels in the eggs of horseshoe crabs (their primary prey) in 2019 and 2022/2023. Figure 3 is on a logarithmic graph because of the large differences within and among the metal levels in laughing gulls. There was a relationship between the mean levels of different elements in the blood of laughing gulls and the mean levels in horseshoe crab eggs, except for As. Arsenic was bioaccumulated at a lower rate in the blood compared to the As in crab eggs.

## 4. Discussion

The main findings of this study were that (1) Hg levels increased from 2019 to 2022/2023, while Cd and Se decreased; (2) there were some significant correlations in the blood of laughing gulls, primarily for As, Cr, Cd, and Pb; and (3) the blood of laughing gulls had similar levels to those found in the eggs of horseshoe crabs. These conclusions informed two other issues: are the levels in laughing gulls similar to those of shorebirds that forage on the same prey, at the same time, in the same locations? And are levels in the blood of laughing gulls at levels that could pose an adverse effect? There are few other studies that have examined the levels of metals in the blood of gulls.

### 4.1. Temporal Differences in Element Levels from 2019 to 2022/2023

In Delaware Bay, laughing gulls accumulated significantly higher levels of Hg and lower levels of Cd and Se in 2022/2023 compared to the levels in 2019. There were no significant temporal differences in the other metals. While declining levels are usually a sign of an environmental improvement, the decline in Se poses a potential risk to the laughing gulls because Se and Hg each mitigate the effect of the other. That is, Se can partly reduce the adverse effect of Hg [45,46], but while Hg increased, Se declined (possibly increasing the impact of Hg on the gulls).

When pooling the data for the sampling periods, there were some highly significant positive correlations among some metals, particularly for As and Cr with other metals (Cd and Pb), which bears further examination, given the prior contamination from chemical plants farther north in the bay [2]. Additionally, Hg was negatively correlated (although the correlations were lower) with Cd and Pb; all three of these elements are contaminants of concern for the health of both biota and humans (see below).

### 4.2. Relationship between Levels in the Blood of Laughing Gulls and Horseshoe Crab Eggs

There was a clear relationship between the mean levels of metals and metalloids in the blood of laughing gulls and the mean levels in their primary prey, providing information on the food chain relationships. It is relatively unusual to have an opportunity to examine the metals in the blood where the birds are feeding primarily on one prey item. For laughing gulls, As was an outlier in that the mean levels of As in their blood were less than those found in horseshoe crab eggs (see Figure 3). This provides useful information to conservationists and regulators that the relationship between the contaminant levels in predators and prey is very tight. It suggests that the sources of the contaminants should be investigated, particularly point-source pollution upstream in the Delaware River [47,48,49].

### 4.3. Comparison of Blood Levels in Laughing Gulls with Those of Shorebirds

One of the illuminating comparisons for birds foraging in Delaware Bay in May is whether the levels of heavy metals in laughing gulls are similar to those of migrant shorebirds when both the gulls and shorebirds were feeding on the same food source (horseshoe crab eggs) at the same time. Comparative data are available for 2019 from Burger [44. Unpub. data]. Laughing gulls had significantly higher levels of Cr and Pb than three species of shorebirds; ruddy turnstones (*Arenaria interpres*) had the highest levels of Cd; and sanderlings (*Calidris alba*) had significantly higher levels of the other metals [13,44]. One unusual finding was that the mean As level in laughing gulls was an order of magnitude lower than that in the blood of shorebirds. The lowest mean level of As in the blood of any shorebird in 2019 was 1690 ± 141 ppb, found in red knots, while for laughing gulls in the same year, it was 452 ppb [13]. Although the gulls were concentrated on Reeds Beach and nearby beaches, the shorebirds ranged farther south and farther north on the bay beaches. Laughing gulls, however, had significantly higher levels of Cd and Pb and significantly lower levels of As than the gulls. This finding was unexpected. We had predicted that the levels would be similar because the gulls and shorebirds eat the same prey, and some gulls remain along the shore feeding all day.

The only clear ecological difference is that, as a group, the shorebirds are exposed for fewer weeks than the gulls, and shorebirds feed almost entirely on eggs. The gulls arrive in April and have several more weeks to feed on crab eggs in the bay, depending on when the horseshoe crab spawning begins. Gulls as a species consume a greater diversity of foods [15]; some metals may have been acquired elsewhere. The differences might reflect different exposure opportunities, unrealized dietary differences, or differences in the toxicokinetics between gulls and shorebirds. Perhaps earlier in the season (when shorebirds had not yet arrived), there were higher levels of Cd in the crab eggs than later, or the eggs themselves had different levels of metals earlier in the season. This latter idea requires examination. Although extensive analyses have been completed on crab eggs from Delaware Bay [45,46], no attempt has been made to examine seasonal differences.

A possible explanation for why As was so much lower is unclear, unless the source of As in the blood collected from the shorebirds was from shorebirds feeding farther north along the Jersey side of the bay, where most of the laughing gulls do not normally forage. This may be a possibility, since from the 1940s to the 1980s, an herbicide plant on the Maurice River released As compounds into the water [50]. The shorebirds feed all along the shore from the Cape Shore Lab up to Gandy’s Point and Money Island. The gulls forage primarily along Villas to Reeds Beach because it is directly across from Stone Harbor, where the gulls nest on the coastal salt marshes. Since there are sufficient crab eggs for the gulls, they do not fly farther afield. Thus, they might be eating crab eggs with a particular metal composition, as the metal levels vary along the Delaware Bay shoreline [2].

### 4.4. Comparison of the Levels of Metals and Metalloids in the Blood of Laughing Gulls with Other Gulls

While there are many papers that examine the levels of metals in the feathers of birds, very few examine the levels of metals in the blood of birds, let alone gulls. Still, in the 1990s, herring gulls (*Larus argentatus*) in nearby Long Island were examined, and Franklin’s gulls *(Larus pipixcan*) were examined in Minnesota [51]. The metals were generally in the same order of magnitude, except for As and Hg (within one order of magnitude), and Se, which was two orders of magnitude higher in laughing gulls than the level of Se in the blood of the other two species tested 30 years earlier (Table 3).

### 4.5. Consequences

The potential consequences of the metals in laughing gull eggs are important because the gulls consume prey that is directly contributing to the production of their own eggs. The gulls foraging on horseshoe crab eggs comprise the population breeding in the marshes across the peninsula on the salt marshes in Stone Harbor [18]. Laughing gulls lay eggs in mid-May to early June, so the numbers of gulls on the beaches begin to decrease when they lay eggs because one member of the pair remains at the nest, first to guard the first-laid eggs, and then to incubate. Non-incubating and non-breeding gulls continue to forage on the horseshoe crab eggs at least into mid-June, although the numbers decline.

The most important metals in terms of toxicity for vertebrates are Cd, Pb, and Hg, which are naturally occurring in saltwater [1,52,53,54,55]. Hg and Pb are especially toxic to vertebrates, including humans. Pb in the environment comes from the former use of Pb paint, gasoline, and lead arsenate insecticides used in agriculture [53,56], while Hg comes from natural sources (volcanoes, the ocean) as well as emissions from coal-fired power plants, industrial sites, and gold mining [57,58,59]. Both Pb and Hg can cause neurobehavioral effects and physiology deficits, and alter reproductive success [57,60,61]. Although Cd is known to be toxic, very few data are available that are relevant to birds [53].

Determining the threshold for adverse effects is difficult because, for most metals and metalloids, there are few laboratory studies that have shown which levels of these elements in the blood are associated with adverse effects. However, some sublethal-effect levels have been reported. For example, a sublethal-effect level reported for Cd in the blood of birds is 260 ppb [62]. All the analyzed samples of the blood of laughing gulls had levels below this presumed toxic level, suggesting no cause for concern. Pb, on the other hand, causes several sublethal effects, as well as direct mortality [63]. Although there are many studies on Pb effects, it is difficult to determine the sublethal effects for gulls. For example, Eisler [53] suggested that adverse effects occur at 4000 ppb, while Franson [64] reported 200 ppb as a toxic threshold for Pb in the blood of birds. In this study, laughing gulls averaged above 300 ppb, but were well below 4000 ppb, which is also the sublethal threshold for behavioral deficits in locomotion, balance, foraging behavior, and parental and sibling recognition in herring gulls [65,66]. It is likely that the laughing gulls do not experience deficits due to Pb.

In vertebrates, including humans, the methylmercury (MeHg) form of Hg poses the greatest risk [55,67,68]. Developing embryos and young are the most vulnerable. Hg can affect avian behavior, physiology, and reproductive success [57,61]. Unlike many of the other metals, the reproductive and neurobehavioral effects of Hg on birds have been clearly demonstrated in the laboratory and in the field [69,70,71,72]. The low-threshold risk for Hg in avian blood is 200 ppb; high-risk levels are 3000–4000 ppb [73]. The laughing gulls in this study averaged Hg blood levels of 473 ppb in 2022/2023, which suggests a need to determine the adverse effects of mercury levels in shorebirds.

While Se occurs naturally in some soils, it is the Se released from soil during the drainage of irrigation water that has posed the greatest toxic problem for birds [45,74,75,76]. Although it is not often measured in the tissues of birds, it is important because it is an essential element with a very narrow range of acceptable levels (e.g., it is toxic at both low and high levels) [74]. However, it can partly ameliorate the toxic effects of Hg [45,46,77]. Importantly, in this study of blood from laughing gulls, the Se levels declined as Hg increased, decreasing the potential protective effects of Se on Hg.

Although there are few laboratory studies of the effects at particular blood levels, Yamamoto and Santolo [78] found that levels of 1500 ppb in adult American kestrels (*Falco sparverius*) resulted in a reduced body condition. The levels of Se in laughing gulls in the present study averaged 18,470 ppb in 2019, but only 5607 ppb in 2022/2023, which is a substantial decrease, suggesting that further investigation is essential, but both are above the effect level (e.g., they are above Yamamoto and Santolo’s [78] level), but similar to the level of 17,000 ppb that caused weight loss in eiders (*Somateria mollissima*)) [79]. It is unclear whether gulls are affected at these high levels.

The levels of As in the blood of laughing gulls (overall mean of 443 ppm) were well below the effect level of 2000 ppb [80,81], and were significantly lower than the levels in shorebirds [13], which is unusual because they feed on the same horseshoe crab eggs. There is potentially higher As in the northern Delaware Bay beaches where shorebirds forage, due to legacy As in the Maurice River from chemical plants [2]. Although there are fewer laughing gulls that forage on the northern beaches, there are some, and an examination of the levels of As in the blood of gulls might indicate higher levels in the gulls from these beaches. This would also require some site-specific testing of horseshoe crab eggs in connection with sampling the blood of laughing gulls. Cr is another metal not usually analyzed in the blood of birds, and there are no laboratory studies that have established effect levels.

## 5. Conclusions

Laughing gulls and several species of shorebirds feed on the eggs of horseshoe crabs in the spring when the crabs breed. Like shorebirds, laughing gulls forage mainly on horseshoe crab eggs, but they do so in close proximity to their breeding colony in Stone Harbor, while the shorebirds are refueling for their migratory journey thousands of km north. Except for As, the mean levels of metals and metalloids in the blood of laughing gulls were correlated with those in the eggs of the crabs. Further, the Hg levels increased in the blood of laughing gulls, and the levels of Cd and Se declined from 2019 to 2022/2023. A significant proportion of laughing gulls had levels of Hg and Se in their blood that were above the known effect levels in birds, suggesting the need for further research. Moreover, since Se partly mitigates the effects of Hg, the decrease in Se while Hg increased in blood might pose an additional risk of adverse effects from Hg. Even without considering any mitigating effects from Se, most laughing gulls in 2022/2023 had levels of Hg that were above the effect level (refer back to Figure 2).

## Figures and Tables

**Table 1:** Metal levels in ng/g (ppb wet weight) in the blood of laughing gulls foraging on horseshoe crab eggs in Delaware Bay in May 2019 (n = 18) and in 2022/2023 (n = 28). Given are means ± standard errors, geometric means, and ranges. Statistics are given in Figure 2.

Metal	2019		2022/2023	
	Mean ± SE (Geometric Mean)	Range	Mean ± SE (Geometric Mean)	Range
Arsenic	452 ± 132 (302)	68–2500	437.7 ± 34.3 (413)	235–1200
Cadmium	26.5 ± 7 (10.7)	0–120	5.5 ± 1.0 (4.1)	1.1–28.5
Chromium	249 ± 99.6 (75.8)	0.1–1700	359.4 ± 121.2 (166.3)	25–3300
Lead	350 ± 85 (227)	32–1500	267.1 ± 48.7 (193.8)	59–990
Mercury	136 ± 31.0 (47.6)	0.1–424	473.1 ± 75.6 (368.1)	98.1–1669
Selenium	18,470 ± 2587 (15,340)	3100–45,000	5607 ± 464 (4500)	160–10,000

**Table 2:** Correlation (Kendall tau) among elements in the blood from laughing gulls in May 2019 and 2022/2023 (n = 46). Samples are from gulls foraging on the eggs of horseshoe crabs. *** = *p* < 0.0001; ** = *p* < 0.001; * = *p* < 0.05.

Metal	As	Cd	Cr	Pb	Hg	Se
As	-					
Cd	0.05	-				
Cr	0.43 ***	0.22 *	-			
Pb	0.21 *	0.42 ***	0.36 **	-		
Hg	−0.07	−0.35 **	−0.10	−0.24 *	-	
Se	−0.11	0.22 *	−0.20	0.07	−0.32 *	-

**Table 3:** Comparison of arithmetic mean blood levels of metals and metalloids of laughing gulls from New Jersey, with Franklin’s gulls from Minnesota and herring gulls from Long Island (New York) in the 1990s. Given are means + standard errors. Units are ppb (ng/g) wet weight.

Metal	Laughing Gull NJ 2019, 2022/2023	Franklin’s Gull Minn 1994	Herring Gull NY 1994
Sample number	46	8	11
As	443 ± 55	50 ± 0.5	28 ± 4
Cadmium	13.5 ± 3.1	6 ± 1	16 ± 7
Chromium	316 ± 83	274 ± 24	197 ± 11
Lead	298 ± 43.9	29 ± 10	233 ± 61
Mercury	338 ± 52.8	56 ± 8	93 ± 21
Selenium	10,460 ± 1370	907 ± 193	355 ± 91

## Data Availability

The data are available from the senior author upon request.
